# A novel molecular subtypes and risk model based on inflammatory response-related lncrnas for bladder cancer

**DOI:** 10.1186/s41065-022-00245-w

**Published:** 2022-08-13

**Authors:** Fucai Tang, Jiahao Zhang, Zechao Lu, Haiqin Liao, Chuxian Hu, Yuexue Mai, Yongchang Lai, Zeguang Lu, Zhicheng Tang, Zhibiao Li, Zhaohui He

**Affiliations:** 1 Department of Urology, The Eighth Affiliated Hospital, Sun Yat-sen University, No. 3025, Shennan Zhong Road, Shenzhen, 518033 Guangdong China; 2 The Sixth Clinical College of Guangzhou Medical University, Guangzhou, 511436 Guangdong China; 3 The Second Clinical College of Guangzhou Medical University, Guangzhou, 511436 Guangdong China; 4 The Third Clinical College of Guangzhou Medical University, Guangzhou, 511436 Guangdong China

**Keywords:** Bladder cancer, inflammatory, long noncoding RNA, TCGA, prognosis

## Abstract

**Background:**

Inflammation and long noncoding RNAs (lncRNAs) are gradually becoming important in the development of bladder cancer (BC). Nevertheless, the potential of inflammatory response-related lncRNAs (IRRlncRNAs) as a prognostic signature remains unexplored in BC.

**Methods:**

The Cancer Genome Atlas (TCGA) provided RNA expression profiles and clinical information of BC samples, and GSEA Molecular Signatures database provided 1171 inflammation-related genes. IRRlncRNAs were identified using Pearson correlation analysis. After that, consensus clustering was performed to form molecular subtypes. After performing least absolute shrinkage and selection operator (LASSO) and multivariate Cox regression analyses, a risk model constructed based on the prognostic IRRlncRNAs was validated in an independent cohort. Kaplan–Meier (KM) analysis, univariate and multivariate Cox regression, clinical stratification analysis, and time-dependent receiver operating characteristic (ROC) curves were utilized to assess clinical effectiveness and accuracy of the risk model. In clusters and risk model, functional enrichment was investigated using GSEA and GSVA, and immune cell infiltration analysis was demonstrated by ESTIMATE and CIBERSORT analysis.

**Results:**

A total of 174 prognostic IRRlncRNAs were confirmed, and 406 samples were divided into 2 clusters, with cluster 2 having a significantly inferior prognosis. Moreover, cluster 2 exhibited a higher ESTIMATE score, immune infiltration, and PD-L1 expression, with close relationships with the inflammatory response. Further, 12 IRRlncRNAs were identified and applied to construct the risk model and divide BC samples into low-risk and high-risk groups successfully. KM, ROC, and clinical stratification analysis demonstrated that the risk model performed well in predicting prognosis. The risk score was identified as an independently significant indicator, enriched in immune, cell cycle, and apoptosis-related pathways, and correlated with 9 immune cells.

**Conclusion:**

We developed an inflammatory response-related subtypes and steady prognostic risk model based on 12 IRRlncRNAs, which was valuable for individual prognostic prediction and stratification and outfitted new insight into inflammatory response in BC.

**Supplementary Information:**

The online version contains supplementary material available at 10.1186/s41065-022-00245-w.

## Introduction

Bladder cancer (BC) is the ninth most universal malignancy, with nearly 550,000 cases recorded in 2018, and ranks fourteenth in cancer deaths worldwide [1]. Urothelial carcinoma, the common type of BC, mainly consists of two different subtypes according to the depth of tumor invasion, namely, nonmuscle-invasive bladder cancer (NMIBC, 70%) and muscle-invasive bladder cancer (MIBC, 20%) [2]. Although NMIBC tends to have a great life expectancy, it frequently recurs (70-80%) and even progresses to MIBC [3]. Facing to future work for therapy of BC, though cisplatin-based chemotherapy is still capital manner, the occurrence of immune checkpoint inhibitors and antiangiogenic therapy further impulse the development of targeted therapy by probing new bio-targets [2]. Therefore, developing a prognostic risk model for bladder cancer patients at the molecular level is critical for assessing risk, identifying novel potential biomarkers, and implementing therapy interventions quickly to improve curative effects and extend patient survival time.

Inflammation has an important protective response by removing stimulants such as alien microorganisms and healing tissue damage [4]. Unfortunately, continuous inflammatory stimulation can lead to chronic inflammation, poor tissue regeneration, tumorigenesis, and metastasis [5]. Pro-inflammatory cytokines are released by tumor-associated inflammatory cells, such as IL-1, IL-6, TNF, and VEGF, which further influences the tumor progression and metastasis [6, 7]. Besides, inflammation has an osculating link with the development and malignant progression of most cancers by regulating DNA damage and repair, p53 mutation, chemokines, and soon [8]. Acute inflammatory response induced by radiotherapy tends to induce an anti-tumor immune response [9]. In contrast, chronic inflammation usually increases tumor occurrence, development, and metastasis by creating a tumor-friendly microenvironment including immunosuppression and angiogenesis [10]. In particular, chronic inflammation can attract a range of immunosuppressive cells, including regulatory T-cells (Tregs), pro-tumorigenic tumor-associated macrophages (TAMs), and myeloid-derived suppressor cells, to develop an immunosuppressive tumor microenvironment (TME) and speed up the formation of tumor [10, 11]. In BC patients, chronic inflammation is one of the risk factors contributing to the tumorigenesis of BC [12]. Increasing pieces of evidence have suggested that chronic inflammation is associated with bladder cancer, and regulating the expression of specific inflammation-related genes can suppress the progression of BC [13–15]. Moreover, predictive significance has been proven for indicators of inflammatory response in BC. C-reactive protein has been shown to be a predictive factor for BC patients' survival by Hilmy M et al [16]. In patients with NMIBC, the lymphocyte-monocyte ratio before surgery is an important predictor of recurrence and development [17]. As a result, a greater knowledge of the link between inflammation and tumor development might aid the study of novel tumor-fighting therapies in BC patients.

LncRNAs participate in the regulation of gene expression via various mechanisms, including interacting with DNA, RNA, or proteins. Moreover, lncRNAs mediate translation control, cell cycle regulation, and apoptosis [18]. Previous research has suggested that lncRNAs may influence the immunological microenvironment of tumors, tumor development, metastasis, and recurrence [19]. And lncRNAs serve as a new component of the innate immune response and intervene in inflammatory signaling [20]. Abnormal lncRNA expression plays a role in carcinogenesis by interfering with biological processes, such as the redirection of chromatin remodeling complexes [21]. To date, the mechanisms of regulating lncRNAs to affect the development of BC have been explored generally in more fields, such as inflammation, self-renewal, and chemoresistance [22, 23]. However, the potential value of lncRNAs concerning inflammation in BC prognosis and treatment has not been made clear until now.

In this study, we retrieved BC patient transcriptome profiles and clinicopathological data from publicly accessible databases (TCGA), and inflammatory response-related lncRNAs (IRRlncRNAs) were screened out by using a series of bioinformatics methods. We employed these IRRlncRNAs to establish inflammatory response-related subtypes based on consensus clustering in BC patients. In addition, after performing Cox regression analysis, candidate IRRlncRNAs were identified to develop a prognostic risk model. Relationships between subtypes, or risk scores and clinicopathological characteristics, function enrichment analysis, and immune microenvironment analysis were investigated further. In summary, the current study may contribute to the exploration of BC prognostic IRRlncRNAs and shed new insights into the potential molecular mechanisms between BC development and inflammatory response.

## Materials and methods

### Data extraction and processing

The specific research process was shown in Additional file 1. We accessed the RNA expression profile and clinical information of 433 BC samples from the TCGA database (https://ocg.cancer.gov/programs/TCGA, accessed on 18 September 2021), which consists of 414 BC samples and 19 normal samples. Inflammatory response-related genes were retrieved from 4 inflammatory response-related gene sets in the Gene Set Enrichment Analysis (GSEA) Molecular Signatures database, including HP_ABNORMAL_INFLAMMATORY_RESPONSE, WP_INFLAMMATORY_RESPONSE_PATHWAY, HALLMARK_INFLAMMATORY_RESPONSE, and MODULE_76 (http://www.gsea-msigdb.org/gsea/msigdb/search.jsp, accessed on 18 September 2021). The additional information on inflammatory response-related genes was saved in Additional file 2.

### Identification of differentially expressed prognostic IRRlncRNAs

Pearson’s correlation analysis was executed between inflammatory response-related genes and all lncRNAs to identify IRRlncRNAs, with the standard of |R| > 0.5 and *P* < 0.01. Differentially expressed IRRlncRNAs between tumor and normal samples were obtained by using the “limma” R package with |log (Fold Change) | > 1 and false discovery rate (FDR) < 0.05. Then, prognostic IRRlncRNAs with *P* < 0.05 were discerned by univariate Cox regression analysis using the R package “survival”.

### Consensus clustering

Founded on the expression profile of prognostic IRRlncRNAs, the “ConsensusClusterPlus” R package was adopted to cluster BC samples into molecular subtypes. We utilized the “Partitioning Around Medoids” clustering algorithm, with each sampling time 85% and repeated one thousand times. The optimum cluster number -- k value was chosen through the cumulative distribution function (CDF) curves and the consensus matrices, which were determined visually, and verified by introducing the proportion of ambiguously clustered pairs (PAC) analysis [24]. Besides, it was also assessed the performance by the t-distributed stochastic neighbor embedding (t-SNE) method.

### Clinical significance, enrichment process, and immune infiltrating analysis of molecular subtypes

Kaplan–Meier (KM) survival analysis was utilized to calculate differences in overall survival (OS) of BC patients between molecular subtypes. Correlation and distribution between the clinicopathologic characteristics and different clusters were shown by a heatmap, including age, grade, smoking status, and stage.

To explore the potential biological processes and pathways of the two clusters, gene set variation analysis (GSVA) was executed by the “GSVA” R package, including Gene Ontology (GO) and Kyoto Encyclopedia of Genes and Genomes (KEGG) gene sets [25]. Subsequently, GSEA was further executed on hallmark gene sets among subtypes in the GSEA software.

To study the influence of IRRlncRNAs on the TME of BC, the ESTIMATE algorithm was used to evaluate the ESTIMATE score, immune score, and stromal score of each sample by functioning the R package “ESTIMATE” [26]. Next, we adopted CIBERSORT algorithms to gauge and compare the abundance and difference of 22 different immune cells in the inflammatory response-related subtypes [27].

### Construction and validation of the risk model based on prognostic IRRlncRNAs

After removing incomplete survival information samples, 406 BC samples were randomly dismantled into two groups: the training and test cohort at 1:1. The clinical characteristics were presented in Table 1. Based on prognostic IRRlncRNAs, LASSO analysis, which can avoid overfitting and reduce the multicollinearity effect among IRRlncRNAs, was performed with the “glmnet” R package to minimize IRRlncRNAs by making the coefficients of irrelevant IRRlncRNAs comparatively zero and excluded in the training cohort. The optimal penalty parameter for LASSO was ascertained based on the minimum partial likelihood deviance calculated by the ten-fold cross-validation method. Subsequently, a multivariate Cox regression, using these IRRlncRNAs determined by LASSO analysis and the lowest value of the Akaike information criterion (AIC), was applied to sift optimal risk IRRlncRNAs as novel signatures and construct a risk model. In addition, stepwise regression with both directions was used to find the minimum AIC value in the multivariate Cox regression. In the LASSO and multivariate Cox regression, independent variables were the expression matrix of candidate IRRlncRNAs, while response variables were the OS and status of BC patients. The risk score of each sample was determined by the calculation formula as follows: risk score = ∑ (Coefn * Expn).

**Table 1:** Baseline characteristics of patients with BC

Covariates	Total	Train	Test	chi	***P***
Age					
<=65	160(39.41%)	83(40.69%)	77(38.12%)	0.280	0.597
>65	246(60.59%)	121(59.31%)	125(61.88%)		
Gender					
Female	107(26.35%)	62(30.39%)	45(22.28%)	3.444	0.063
Male	299(73.65%)	142(69.61%)	157(77.72%)		
Grade					
High	383(94.33%)	191(93.63%)	192(95.05%)	0.780	0.377
Low	20(4.93%)	12(5.88%)	8(3.96%)		
unknow	3(0.74%)	1(0.49%)	2(0.99%)		
Smoked					
NO	109(26.85%)	63(30.88%)	46(22.77%)	3.790	0.052
YES	284(69.95%)	133(65.2%)	151(74.75%)		
unknow	13(3.2%)	8(3.92%)	5(2.48%)		
Stage					
I-II	131(32.27%)	68(33.33%)	63(31.19%)	0.214	0.644
III-IV	273(67.24%)	135(66.18%)	138(68.32%)		
unknow	2(0.49%)	1(0.49%)	1(0.5%)		
T					
T0/X	2(0.49%)	0(0%)	2(0.99%)	2.683	0.221
T1-2	121(29.8%)	66(32.35%)	55(27.23%)		
T3-4	251(61.82%)	123(60.29%)	128(63.37%)		
unknow	32(7.88%)	15(7.35%)	17(8.42%)		
M					
M0/X	392(96.55%)	196(96.08%)	196(97.03%)	0.088	0.766
M1	11(2.71%)	6(2.94%)	5(2.48%)		
unknow	3(0.74%)	2(0.98%)	1(0.5%)		
N					
N0/X	272(67%)	140(68.63%)	132(65.35%)	0.507	0.477
N1-3	128(31.53%)	61(29.9%)	67(33.17%)		
unknow	6(1.48%)	3(1.47%)	3(1.49%)		

The regression coefficient of each computed lncRNA is Coefn, and the expression level of each computed lncRNA is Expn. According to risk scores, BC samples were split into “high-risk” and “low-risk” groups based on the medium of risk score as the cut-off point in the training cohort and test cohort. KM method also served to assess the prognostic value of the two groups by the “survminer” R package. The performance of the risk model was proven by time-dependent receiver operating characteristic (ROC) curves of 1-, 3- and 5-year survival rates via the “survivalROC” package.

### Clinical stratification analysis of the IRRlncRNA signature

Univariate and multivariate Cox regression was utilized to explore provided the risk score constructed by the prognostic IRRlncRNAs can serve as an independent prognostic factor compared with age, smoked, stage, gender, and risk score as included factors. Further, we conducted a stratified analysis between the risk model and different subgroups with various clinical traits. Specifically, the differences of risk scores were explored in different clinical subgroups using the Wilcoxon test and the “ggpubr” R package. KM survival analysis was applied on investigating the OS distribution in risk groups of clinical stratified variables including smoked history, stage (I-II, III-IV), age (≤65, >65), clustering subtypes, sex, T stage (T1-2, T3-4), M stage (M0, M1), and N stage (N0, N1–3).

### Functional enrichment and immune cell infiltration analysis with risk scores

To reveal the potential biological processes and cause for survival division between the two risk groups, we conducted GSEA founded on the GO function data set (c5.go.v7.4.symbols) and KEGG pathway data set (c2.cp.kegg.v7.4.symbols) that were downloaded on November 2021 from GSEA database. Furthermore, immune cell infiltration based on CIBERSORT was further probed for correlation with risk scores by Spearman’s test with *p* < 0.05 in order to evaluate the changes of immune cells in inflammatory response-related risk scores of BC patients.

### Statistical analyses

The comparison of baseline characteristics between the training and test cohorts was run by the Chi-square test and Fisher’s exact probability test in SPSS 25. And the Wilcoxon test was used to compare differences in risk scores among the clinical characteristic groups. To create survival curves, the KM plot and the Log-rank test were performed to assess statistically significant differences. Statistical analyses were performed by utilizing R version 4.1.0, and statistical significance was assumed at a two-sided p value of less than 0.05. GSEA was run in the GSEA software v4.1.0, and the enriched gene sets with *p* < 0.05 and FDR < 0.25 were included in the analysis.

## Results

### Screening of IRRlncRNAs in BC

From the GSEA Molecular Signatures database, 1171 inflammatory response-related genes were identified in total (Additional file 2). Then, we obtained 1972 IRRlncRNAs linked with the inflammatory response through the coregulation correlation test (Additional file 3). In tumor (n=414) and normal (n=19) samples, 1086 IRRlncRNAs were differentially expressed, with 336 upregulated lncRNAs and 750 downregulated lncRNAs (Additional file 4). Furthermore, univariate Cox regression was applied to resolve whether 1086 IRRlncRNAs were related to prognosis in BC patients with OS. Among them, 174 IRRlncRNAs were associated with prognosis and included in the following analysis (*P* < 0.05; Additional file 5).

### Consensus clustering to identify subtypes

**Fig. 1:**
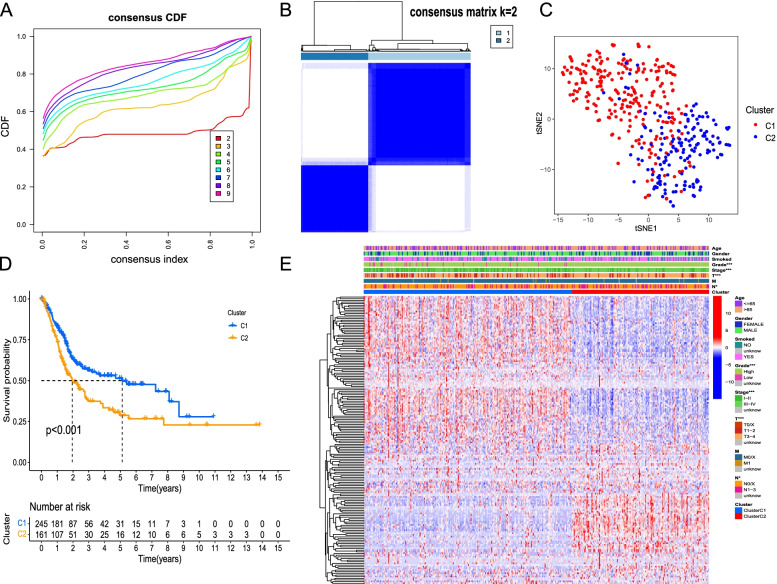
Distribution of clinicopathologic features and prognosis among the two clusters. **A** CDF curves for cluster number k = 2-9. Y axis is CDF value, and X axis is consensus index. **B** Consensus clustering yielded a color-coded heatmap matching the consensus matrix of the two subtypes. **C** t-SNE revealed two unique distribution modes between the two subtypes. **D** Kaplan–Meier analysis of samples in two clusters. **E** Heatmap of the expression of IRRlncRNAs and clinical characteristics among the two subtypes. Each row of heatmap is each IRRlncRNA, and each column is each BC sample

To identify different inflammatory response-related subtypes in BC samples, consensus clustering based on 174 prognostic IRRlncRNAs was undertaken (k = 2 to 9). When k = 2 was carried out, the CDF curve of the consensus index score had a highly steady trend and the flattest slope, and the interference between the 2 clusters was considerably small (Fig. 1A-B). And k = 2 also was verified as the optimal cluster number by PAC method. Therefore, 406 BC samples, in which survival information was available, were subsequently categorized into two subtypes – cluster 1 (n = 245) and cluster 2 (n = 161) (Fig. 1B), and then were visualized successfully by performing t-SNE (Fig. 1C). Kaplan–Meier survival analysis in our study revealed that samples in cluster 1 had a superior prognosis (*P* < 0.001; Fig. 1D). Furthermore, there were substantial differences in clinicopathologic features, including tumor grade, stage, T stage (all *P* < 0.001), and N stage (*P* < 0.05), between cluster 1 and cluster 2 (Fig. 1E). Specifically, in comparison to cluster 1, cluster 2 has a higher proportion in high grade, III-IV stage, T3-T4 stage, and N1-N3 stage of BC patients. In conclusion, inflammatory response-related subtypes have a significant correlation with the clinical heterogeneity of BC samples.

### More active gene set enrichments in cluster 2

**Fig. 2:**
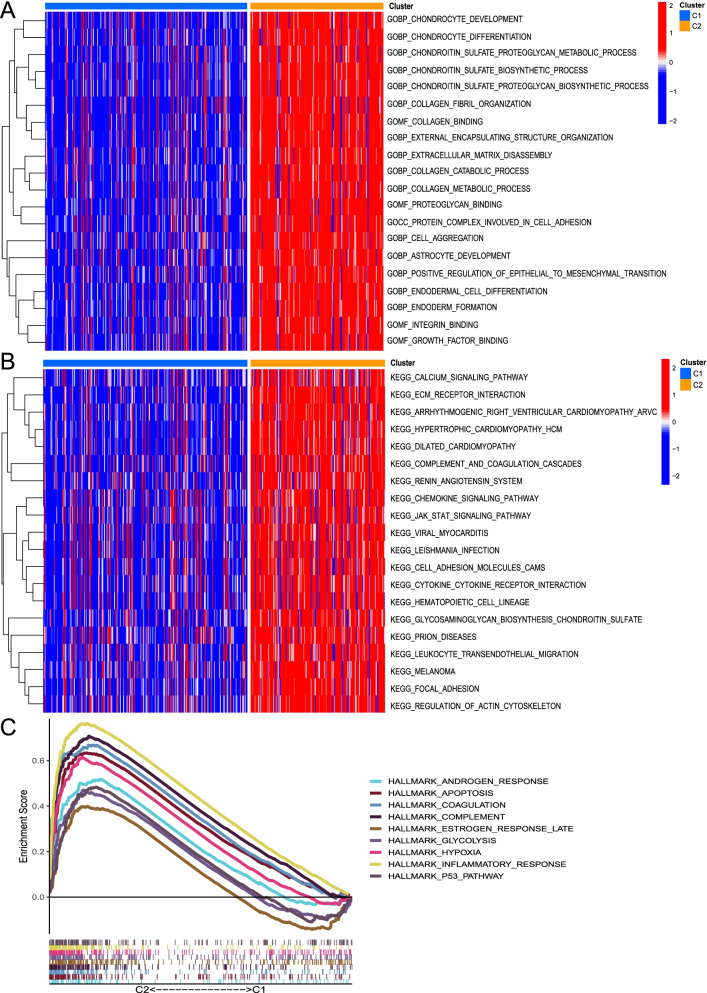
Gene set enrichment analysis in clusters 1 and 2. **A**-**B** Heatmap of the enrichment analysis using the GSVA algorithm based on GO (A) and KEGG (B) gene sets. **C** GSEA based on hallmark gene sets

To visualize the precise signaling pathways and biological functions affected by IRRlncRNAs of the two clusters, we further performed GSVA-GO, GSVA-KEGG, and GSEA-Hallmark analyses. We found that chondroitin and collagen metabolic processes, positive regulation of epithelial to mesenchymal transition (EMT), and extracellular matrix disassembly were significantly enriched in cluster 2 (Fig. 2A). In cluster 2, pathways such as focal adhesion, calcium signaling pathway, extracellular matrix protein (ECM) receptor interaction, JAK-STAT signaling pathway, chemokine signaling pathway, leukocyte transendothelial migration, and other diseases were differentially elevated (Fig. 2B). Furthermore, the GSEA results revealed that signaling pathways such as the inflammatory response, apoptosis, hypoxia, p53 pathway, and sex hormone response were massively concentrated in cluster 2 (Fig. 2C). In summary, cluster 2 was more prone to the inflammatory response in BC patients.

### Immune infiltration analysis in clusters

**Fig. 3:**
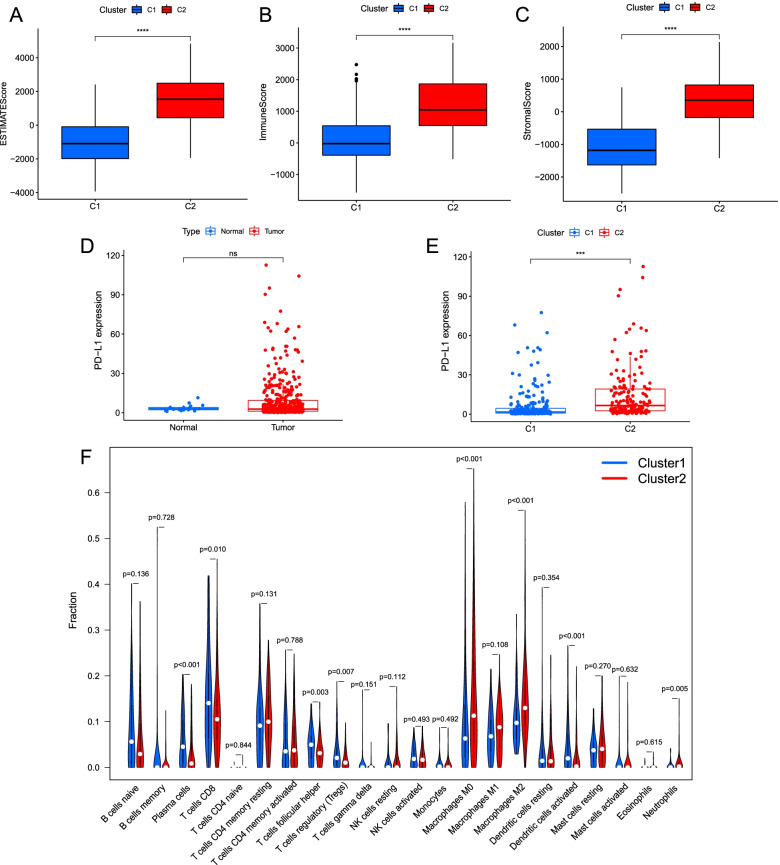
The divisions of inflammatory response-related clusters in the TME of BC. **A-C** The contrast of ESTIMATEScores, StromalScores, and ImmuneScores between two clusters. **D-E** Box plot of the PD-L1 expression differences (D: tumor and normal samples; E: cluster 1 and cluster 2). **F** The infiltration analysis of 22 immune cells in clusters

Some relationships between immunity and IRRlncRNAs need to be probed to furnish some potential views in immunotherapy probably. Cluster 2 had a higher immune score, stromal score, and ESTIMATE score (all *P* < 0.0001; Fig. 3A-C). The expression of PD-L1 in normal and tumor samples did not differ significantly (Fig. 3D), while in comparison to cluster 1, cluster 2 had PD-L1 expression increased (*P* < 0.001; Fig. 3E). Cluster 1 exhibited a greater degree of plasma cells, CD8 T cells, follicular helper T cells, Tregs, and activated dendritic cells, while higher M0 macrophage and M2 macrophage levels were observed in cluster 2 (all *P* < 0.05; Fig. 3F). Taken together, cluster 2 may be related to chronic inflammation.

### Construction and evaluation of the risk model

**Fig. 4:**
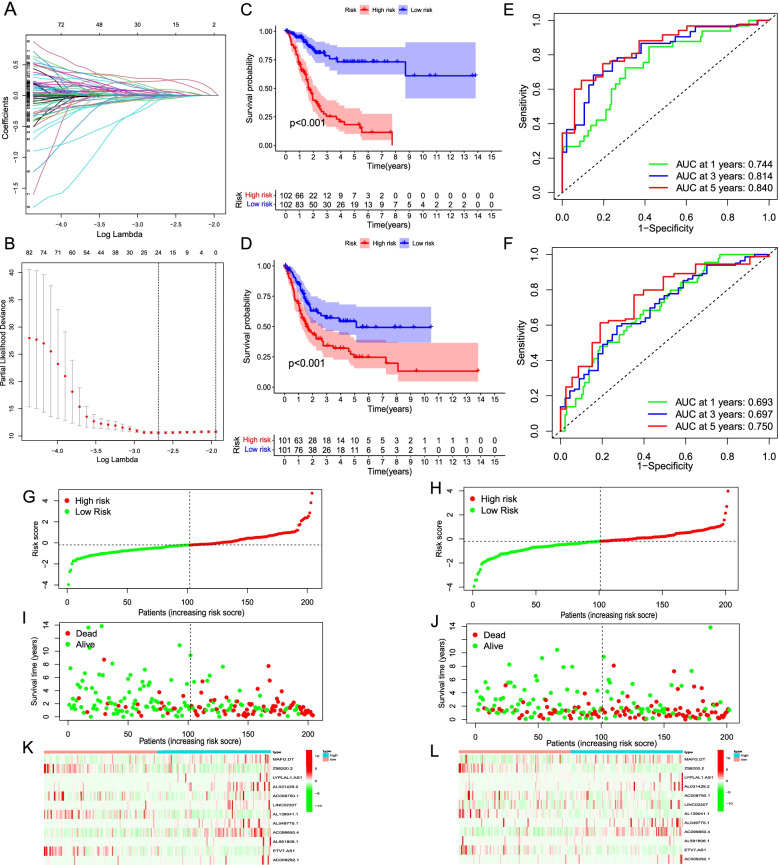
Development of a prognostic risk model based on IRRlncRNAs for BC patients. **A-B** Diagram for LASSO regression analysis based on prognostic IRRlncRNAs. **C-D** Kaplan–Meier plot revealed a notable difference in prognosis between the high- and low- risk groups (C: Training cohort; D: Test cohort). **E-F** Time-dependent ROC analysis represented 1-, 3-, and 5-year predictions based on 12 IRRlncRNAs risk model in BC samples (E: Training cohort; F: Test cohort). **G-H** The risk score curve (G: Training cohort; H: Test cohort). **I-J** Scatter plot of the relationship between survival status and risk score of each sample (I: Training cohort; J: Test cohort). Red dots mean the dead samples, while the green dots mean the alive samples. **K-L** Heatmap revealing the distribution of 12 IRRlncRNAs expression levels in risk groups (K: Training cohort; L: Test cohort)

The above analysis indicated that there are subtypes and regulations based on inflammatory response in BC. Therefore, we constructed a risk score model to further study the prognostic value of IRRlncRNAs in BC. First, we randomly separated 406 BC samples into two cohorts: training (n = 204) and test (n = 202). There were none of statistically significant differences in age, grade, smoked history, gender, stage, or TMN stage between the training and test cohorts (*P* > 0.05; Table 1). Further, based on 174 prognostic IRRlncRNAs, LASSO regression was utilized to reduce the risk of overfitting, and 24 IRRlncRNAs were chosen for further inquiry (Fig. 4A-B). After that, multivariate Cox regression was performed to reckon respective coefficients, resulting in the selection of 12 IRRlncRNAs as risk signatures to develop a risk score model in BC patients (Additional file 6). The following formula was applied to calculate the risk score for BC samples. Risk score = 0.01929269 * expr (MAFG-DT) - 0.486500672 * expr (Z98200.2) + 0.15713061 * expr (LYPLAL1-AS1) + 0.499232227 * expr (AL031429.2) - 0.347294883 * expr (AC008750.1) + 0.358586976 * expr (LINC02207) - 0.263574577 * expr (AL139041.1) + 0.630116014 * expr (AL049775.1) + 0.01165779 * expr (AC099850.4) + 0.071137074 * expr (AL591806.1) - 0.213999812 * expr (ETV7-AS1) + 0.317714659 * expr (AC009292.1).

Based on the medium of risk scores, samples from the training and test cohorts were dismantled into two groups: low-risk and high-risk. And the OS of BC samples in the high-risk group was significantly poorer than that in the low-risk group in the training cohort, which was also consistent in the test cohort (both *P* < 0.001; Fig. 4C-D). Time-dependent ROC demonstrated that the AUCs of 1, 3, and 5 years were 0.744, 0.814, and 0.840, respectively, in the training cohort and 0.693, 0.697, and 0.750, respectively, in the test cohort (Fig. 4E-F). Therefore, the risk model can play an effective role in evaluating the OS of BC patients. Afterward, we computed the risk score of each sample and ranked them by plotting the risk curves (Fig. 4G-H). The scatter plot visualized the relationship between risk scores and survival status of samples in the two cohorts (Fig. 4I-J). The higher the risk scores are, the more samples die easily. Besides, the distribution of 12 IRRlncRNAs expression between two risk groups was exhibited in Fig. 4K-L.

### Clinical stratification analysis of risk scores based on the IRRlncRNA signature

To investigate whether the risk model based on 12 IRRlncRNAs was an independent factor in the prognostic prediction of BC samples, we used univariate and multivariate Cox regression with age, smoked status, stage, gender, and risk score as predictive factors (Table 2). Univariate Cox regression indicated that the risk score was related to the OS and was verified in both the training and test cohorts (both *P* < 0.001). The results of the multivariate analysis suggested that the risk score was an independent predictor of OS in both the training (HR: 2.559, 95% CI: 2.011-3.256, *P* < 0.001) and test cohorts (HR: 1.539, 95% CI: 1.239-1.912, *P* < 0.001).

**Table 2:** Independent prognostic analysis of the riskScore and clinicopathological factors in BC patient set

Variable		Training cohort						Test cohort					
		Univariate Cox			Multivariate Cox			Univariate Cox			Multivariate Cox		
		HR	95%CI	*p*	HR	95%CI	*p*	HR	95%CI	*p*	HR	95%CI	*p*
Age		1.028	1.005-1.053	0.019	1.017	0.994-1.040	0.158	1.040	1.018-1.062	<0.001	1.034	1.012-1.057	0.003
Gender	Male/Female	1.188	0.728-1.939	0.491	1.245	0.747-2.075	0.400	0.571	0.362-0.899	0.016	0.589	0.372-0.931	0.023
Smoked	YES/NO	1.323	0.806-2.171	0.268	0.915	0.538-1.554	0.742	1.170	0.706-1.939	0.542	1.162	0.696-1.939	0.566
Stage	I/II/III/IV	2.168	1.585-2.966	<0.001	1.466	1.051-2.045	0.024	1.526	1.179-1.974	0.001	1.361	1.050-1.763	0.020
riskScore		2.834	2.263-3.550	<0.001	2.559	2.011-3.256	<0.001	1.573	1.309-1.809	<0.001	1.539	1.239-1.912	<0.001

**Fig. 5:**
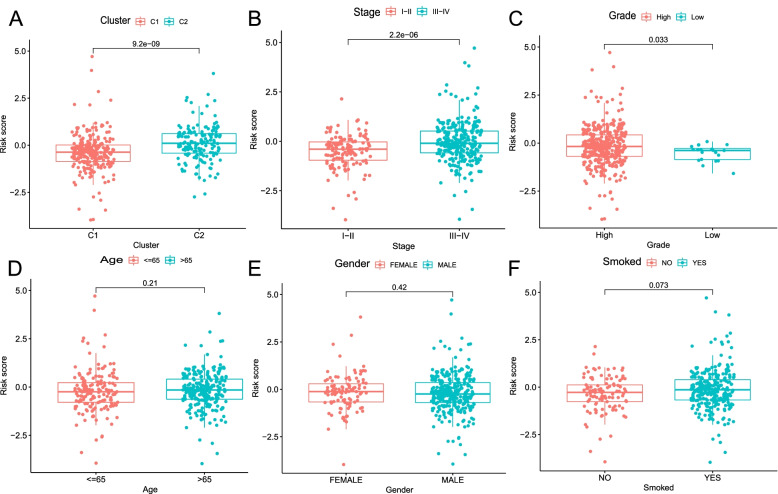
Comparison analysis in risk scores of clinical characteristics. **A** Cluster. **B** Stage. **C** Grade. **D** Age. **E** Gender. **F** Smoked

**Fig. 6:**
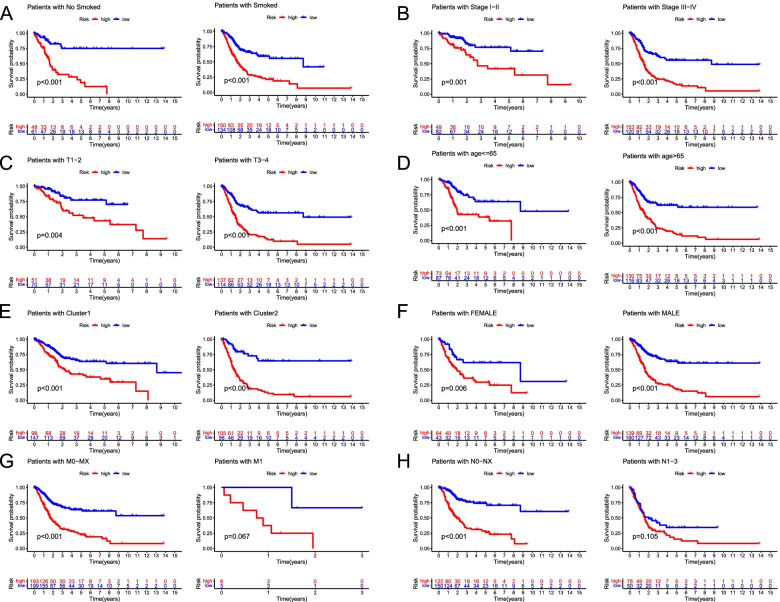
Survival curves for risk groups in clinical stratification. **A** Smoked (No smoked or smoked), **B** Tumor stage (I–II or III–IV), **C** T stage (1-2 or 3-4), **D** Age (<=65 or >65 years old), **E** Cluster (1 or 2), **F** Gender (female or male), **G** M stage (M0-MX or M1), **H** N stage (N0-NX or N1-3)

Further, it was explored that the discrepancy of risk scores in different clinical characteristics. Cluster 2, stage III-IV, and high-grade samples had a higher risk score (all *P* < 0.05), whereas there was no significant link between risk score and age, gender, or smoked (all *P* > 0.05; Fig. 5). Moreover, the relationship between the risk groups and OS was probed to test the prognostic performance of the risk model in different clinical subgroups of BC patients. Samples with a low-risk score had a longer OS than those with a high-risk score in multiple categories, such as smoking, tumor, stage, sex, T stage, and cluster (all *P* < 0.05; Fig. 6A-F). In addition, in subgroups of M and N stages, the OS of M1 and N1-3 BC samples had no statistical significance for those with low-risk scores compared with those with high-risk scores (*P* > 0.05; Figs. 6G-H).

### Signaling pathways in high- and low-risk groups

**Fig. 7:**
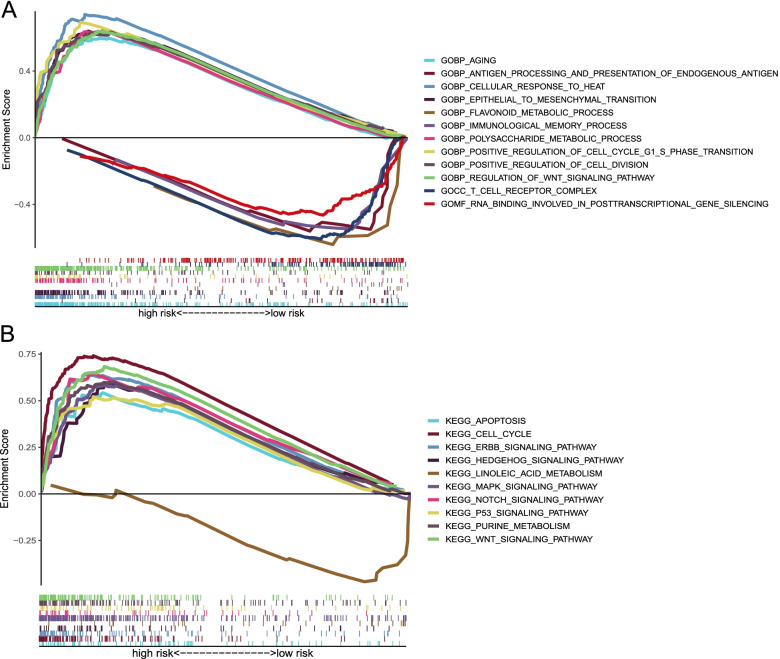
GSEA revealed significant signaling pathway differences between the high- and low-risk groups. **A** GSEA-GO analysis; **B** GSEA-KEGG analysis.

To elucidate the difference in biological function between the two risk groups, we conducted GSEA based on GO and KEGG sets. Immune-related pathways, including immunological memory process, T-cell receptor complex, and antigen processing and presentation of endogenous antigen were enriched in the low-risk group, while EMT was more abundant in the high-risk group (Fig. 7A). Nevertheless, the P53 signaling pathway, MAPK signaling pathway, and WNT signaling pathway were significantly enriched in the high-risk group (Fig. 7B).

### Correlation of the risk score and immune cell infiltration

**Fig. 8:**
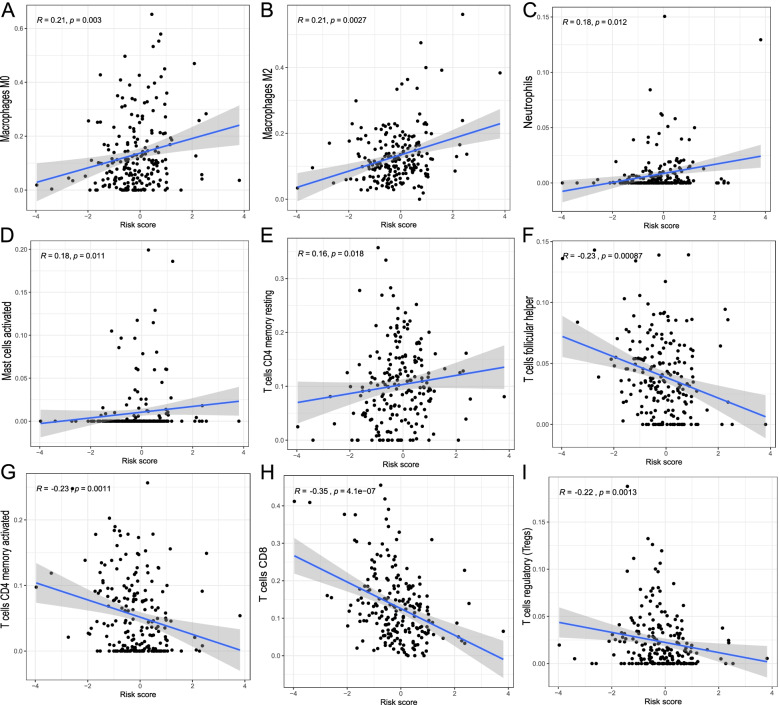
The correlation between the risk score and 9 immune cell types. **A** M0 macrophages, **B** M2 macrophages, **C** neutrophils, **D** activated mast cells, **E** resting CD4 memory T cells, **F** follicular helper T cells, **G** activated memory CD4 T cells, **H** CD8 T cells, **I** regulatory T cells (Tregs)

Since the above GSEA-GO analysis result was connected to immune-related pathways, Spearman correlation analysis was performed on the infiltration of various immune cells in BC samples to observe whether there was a link between the risk score based on the twelve IRRlncRNAs and immune infiltration. The risk score was shown to be positively connected with the infiltration of M0 and M2 macrophages, neutrophils, activated mast cells, and CD4 memory resting T cells, whereas follicular helper T cells, CD4 memory activated T cells, CD8 T cells and Tregs were negatively associated with the risk score (all *P* < 0.05; Figs. 8A-I).

## Discussion

Bladder cancer is a heterogeneous and strong metastatic disease, with approximately 170,000 deaths every year [27]. According to global cancer statistics in 2018, bladder cancer accounts for 3.0% of all newly diagnosed cancer cases and 2.1% of all cancer deaths [28]. Recently, since high-throughput biological technology has been applied to assist in early disease diagnosis and the discovery of therapeutic targets, we are eager to provide a more reliable and accurate prognostic risk assessment method for BC patients to predict survival and probe potential help for improving clinical treatment strategies based on the inflammatory response.

For exploring more potential inflammatory response-related targets and mechanisms in development of tumor, similar risk models were successfully constructed in many cancers, such as nephroblastoma and bladder cancer [29, 30]. Inflammatory diseases are linked to lncRNAs, which might be used as biomarkers to diagnose inflammatory diseases such as rheumatoid arthritis [31]. Previous studies have mainly focused on urinary biomarkers in diagnosis and monitoring, such as urine DNA methylation assay, while inflammatory biomarkers in the blood have also recently been demonstrated to be potential biomarkers in prognosis of BC patients, such as plasma fibrinogen and D-dimer [32–34]. Currently, a prognostic risk model based on the inflammatory response associated with mRNA may effectively distinguish BC patients with a good or worse prognosis [30]. IRRlncRNAs, on the other hand, have yet to be identified as predictive markers for BC. In comparison to these risk models described above, the risk model of our study based on IRRlncRNAs developed offered higher benefits in BC patients.

Consensus clustering has been widely performed to preliminarily discover more potential molecular subtypes in BC, such as clusters of hypoxia response, m6A-related, and immune-related [35–37]. Similarly, using consensus clustering, our research effectively demonstrated that there are inflammatory subtypes based on IRRlncRNAs in BC patients. Our study hopes to determine potential reasons and mechanisms by GSEA and immune differences analysis in the prognosis and development of BC. The results showed that in addition to the inflammatory response, cluster 2 was also linked to EMT, ECM receptor interactions, the p53 pathway, and hypoxia. A recent study revealed that EMT can progress the migration and development of tumors by regulating various lncRNAs [38]. Moreover, it was proved that EMT can be enhanced to boost tumor metastasis by HSF1 combined with LEF1 dependence in BC [39]. ECM receptor interactions have a wide impact on tumor cell behaviors. Zhang H et al found that ECM is linked to the progression of NMIBC to MIBC patients through the NF-κB and PI3K/Akt signaling pathways [40]. Besides, regulating the p53 pathway by lncRNA LOC572558 can repress proliferation in bladder cancer [41]. Hypoxia, an indispensable contributor to tumor development, the results in a significant number of inflammatory factors and then triggers macrophage polarization that transforms M1 (proinflammatory and antitumor) cells into M2 (anti-inflammatory and protumor) cells [42]. In summary, the above pathway analysis may serve as an explanation for the poor prognosis of cluster 2.

LncRNAs have been discovered to be involved in a variety of physiological systems and illnesses. To identify a novel prognostic model and study its clinical value in BC, we used the training cohort to develop a novel risk model based on 12 IRRlncRNAs as a prognostic signature and used the test cohort to validate the reliability. The model in this study comprised 12 IRRlncRNAs (MAFG-DT, Z98200.2, LYPLAL1-AS1, AL031429.2, AC008750.1, LINC02207, AL139041.1, AL049775.1, AC099850.4, AL591806.1, ETV7-AS1, AC009292.1). Among them, Z98200.2, AC008750.1, AL139041.1, and ETV7-AS1 were shown to be protective of BC prognosis (Hazard ratio < 1 and Coef > 0), while MAFG-DT, LYPLAL1-AS1, AL031429.2, LINC02207, AL049775.1, AC099850.4, AL591806.1, and AC009292.1 were identified as risk factors for BC prognosis (Hazard ratio > 1 and Coef > 0, Additional file 6). We found that MAFG-DT expression was negatively connected with BC prognosis in our investigation, which was similar to the findings of Zheng Z et al [43]. The link between AC008750.1 and NK cells was studied by Sage et al, who revealed that the expression of AC008750.1 was elevated in activated NK cells, and antitumor capabilities of NK cells can be repressed when knocking down the expression of AC008750.1 [44]. In addition, AL591806.1 and LINC02207 are also potential immune-associated lncRNA signatures in BC and other cancer [45, 46]. And LYPLAL1-AS1 has also been verified to engage in the adipogenic differentiation and senescence of human stem cell [47, 48]. Therefore, these IRRlncRNAs identified by our study can participate in other biological processes probably. It needs to note that molecular subtypes and the relationship between 12 IRRlncRNAs and the inflammatory response still need further study by experiments in BC.

Because of its critical involvement in tumor-antagonizing or tumor-promoting actions, the tumor immune microenvironment has received much attention. In our study, the results of immune infiltration analysis suggested that there was immune heterogeneity within the two clusters. Samples of cluster 2 had increased ESTIMATE scores, immune scores, and stromal scores. Besides, cluster 2 also tended to have more infiltration of M0 and M2 macrophages, and these macrophages related positively to risk scores of BC. Recent studies have suggested that macrophages predominate in chronic inflammation and the myeloid cell of tumors, which is generally considered to promote malignancy, immunosuppression, and metastasis [49, 50]. It was reported that TAMs are thought to exert an immunosuppressive effect by preventing CD8^+^ T cells from the antitumor immune response [51]. And our study also found that the greater risk scores are, the lower CD8^+^T cells infiltration. And cluster 2 also has lower CD8+T cells infiltration and higher PD-L1 expression. It is known that PD-L1 can inhibit the activation of CD8^+^T cells. When the activation of PD-L1 was blocked using relevant inhibitor, immune escape can be repressed in BC [52]. Therefore, anti-PD-L1 therapy may be more beneficial to cluster 2 of BC patients in our patients. Qiu et al. demonstrated that macrophages regulate the progression of collagen and PI3K/AKT signaling pathway to stimulate BC cell growth [53]. Coincidentally, GSVA-GO indicated that collagen-associated pathways were enriched in cluster 2. As a result, we may reasonably assume that macrophages of the TME promote the progression of tumors in cluster 2 via collagen-associated pathways and participate in the occurrence of chronic inflammation, which may result in a poor prognosis.

However, there are a few limitations to this study that should be considered. This research needs further molecular biology experiments focused on inflammatory response-related lncRNAs in BC. And the number of IRRlncRNAs to construct the risk model may influence clinical usability of the risk model. In addition, our study only used the OS to analyze the prognostic value in survival analysis without consideration of other potential confounding factors, such as surgical interventions, owing to relevant information being incomplete in TCGA datasets. Ultimately, the 12-IRRlncRNA signature was only verified in the TCGA validation cohort, because none of the datasets (ICGC and GEO) had a total of 12 risk IRRlncRNAs to construct and validate our risk model adequately.

## Conclusion

In summary, 2 inflammatory response-related subtypes based on IRRlncRNAs were successfully distinguished and established to find potential role of inflammatory response in BC. Further, we also successfully developed a predictive risk model based on 12 IRRlncRNAs as prognostic signatures, which can serve as an independent prognostic factor for BC patients. In addition, novel IRRlncRNA signatures may predict the prognosis of BC patients and make novel insight into further therapy regimens combined with the development of an inflammatory response in BC.

## Supplementary Information

**Additional file 1.** The specific research flow process.

**Additional file 2.** Additional annotations of inflammatory response-related genes.

**Additional file 3.** The co-expression analysis results of inflammatory gene and lncRNA.

**Additional file 4.** The results of differentially expressed IRRlncRNAs.

**Additional file 5.** Prognostic IRRlncRNAs obtained by univariate Cox regression.

**Additional file 6.** The coefficient, hazard ratio, and gene ID of 12 IRRlncRNAs in the multivariable Cox regression.

## Data Availability

The datasets supporting the conclusions of this article were included within the article and its additional files. Specifically, the RNA-seq data sets and patient clinical characteristics were downloaded from the TCGA database (https://ocg.cancer.gov/programs/TCGA). The inflammatory response-related gene sets were obtained from the GSEA database (http://www.gsea-msigdb.org/gsea/msigdb/search.jsp). These databases were publicly open and accessed.

## Abbreviations

BC: Bladder cancer
CDF: Cumulative distribution function
ECM: Extracellular matrix protein
EMT: Epithelial-mesenchymal transition
FDR: False discovery rate
GO: Gene Ontology
GSEA: Gene Set Enrichment Analysis
GSVA: Gene set variation analysis
lncRNAs: Long noncoding RNAs
IRRlncRNAs: Inflammatory response-related lncRNAs
KEGG: Kyoto Encyclopedia of Genes and Genomes
LASSO: Least absolute shrinkage and selection operator
MIBC: Muscle-invasive bladder cancer
NMIBC: Nonmuscle-invasive bladder cancer
OS: Overall survival
ROC: Receiver operating characteristic curve
TAMs: Tumor-associated macrophages
TCGA: The Cancer Genome Atlas
TME: Tumor microenvironment
Tregs: Regulatory T-cells
t-SNE: T-distributed stochastic neighbor embedding
