# Supplementary figures and images for: A novel molecular subtypes and risk model based on inflammatory response-related lncrnas for bladder cancer
Source: Hereditas. 2022 Aug 13;159:32. doi: 10.1186/s41065-022-00245-w (PMC9375404; doi:10.1186/s41065-022-00245-w)

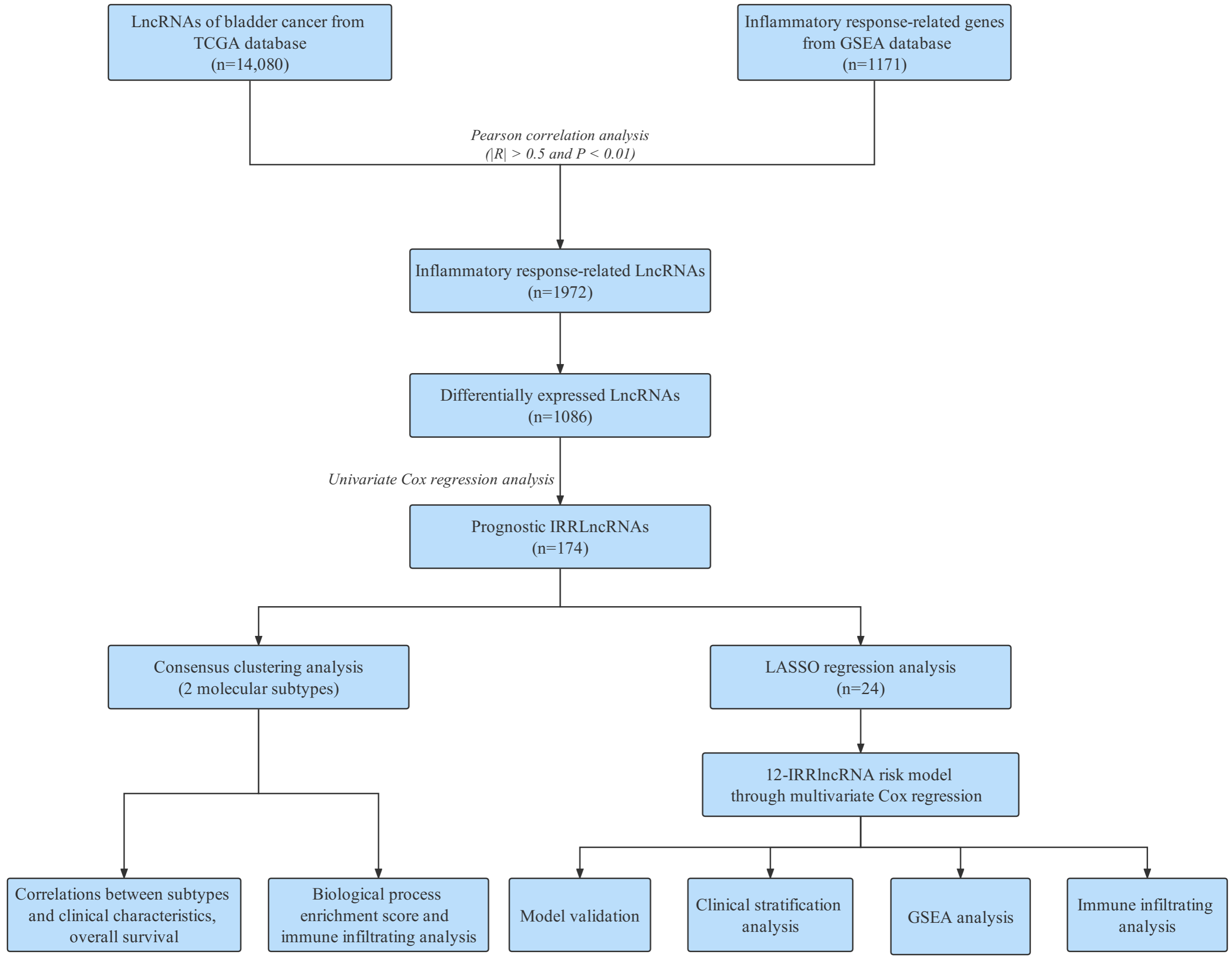

Supplement: Supplementary file 1 — Additional file 1. The specific research flow process. [file 41065_2022_245_MOESM1_ESM.pdf]
